# Bis(benzyl phenyl sulfoxide-κ*O*)dichloridodiphenyl­tin(IV)

**DOI:** 10.1107/S1600536809039269

**Published:** 2009-10-03

**Authors:** Yan-Qiu Dang

**Affiliations:** a Department of Chemistry & Chemical Engineering, Binzhou University, Binzhou 256600, People’s Republic of China

## Abstract

The mol­ecule of the title compound, [Sn(C_6_H_5_)_2_Cl_2_(C_13_H_12_OS)_2_], has crystallographic twofold symmetry. The Sn^IV^ atom is six-coordinate within a distored octa­hedral geometry defined by a C_2_Cl_2_O_2_ donor set.

## Related literature

For general background to organotin compounds and their applications, see: Davies (2004 ▶); Tian *et al.* (2005 ▶); Hadjikakou & Hadjiliadis (2009 ▶). For related structures, see: Ng & Rheingold (1989 ▶); Boa *et al.* (1995 ▶); Tian *et al.* (1998 ▶); Sadiq-ur-Rehman *et al.* (2007 ▶).
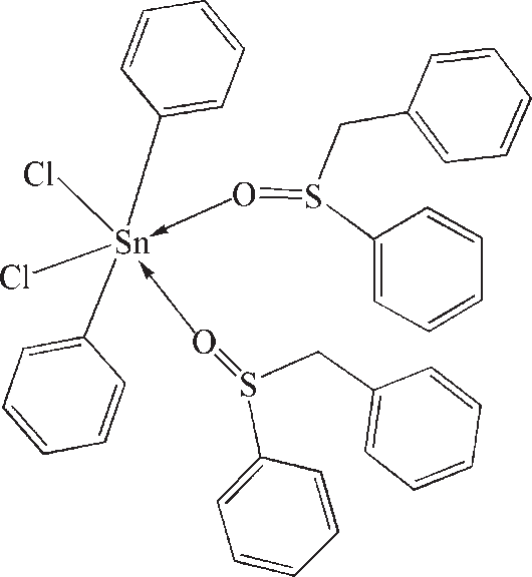


## Experimental

### 

#### Crystal data

[Sn(C_6_H_5_)_2_Cl_2_(C_13_H_12_OS)_2_]
*M* *_r_* = 776.40
Monoclinic, 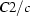

*a* = 22.7485 (19) Å
*b* = 11.5478 (14) Å
*c* = 16.984 (2) Å
β = 126.633 (6)°
*V* = 3580.3 (7) Å^3^
*Z* = 4
Mo *K*α radiation
μ = 1.01 mm^−1^
*T* = 295 K
0.25 × 0.22 × 0.11 mm

#### Data collection

Bruker SMART APEX area-detector diffractometer
Absorption correction: multi-scan (*SADABS*; Bruker, 2002 ▶) *T*_min_ = 0.786, *T*_max_ = 0.897
9821 measured reflections
3512 independent reflections
3003 reflections with *I* > 2σ(*I*)
*R*_int_ = 0.031

#### Refinement

*R*[*F* ^2^ > 2σ(*F* ^2^)] = 0.032
*wR*(*F* ^2^) = 0.076
*S* = 1.03
3512 reflections
204 parameters
H-atom parameters constrained
Δρ_max_ = 0.43 e Å^−3^
Δρ_min_ = −0.37 e Å^−3^

### 

Data collection: *SMART* (Bruker, 2002 ▶); cell refinement: *SAINT* (Bruker, 2002 ▶); data reduction: *SAINT*; program(s) used to solve structure: *SHELXS97* (Sheldrick, 2008 ▶); program(s) used to refine structure: *SHELXL97* (Sheldrick, 2008 ▶); molecular graphics: *ORTEP-3* (Farrugia, 1997 ▶); software used to prepare material for publication: *SHELXL97*.

## Supplementary Material

Crystal structure: contains datablocks global, I. DOI: 10.1107/S1600536809039269/tk2538sup1.cif

Structure factors: contains datablocks I. DOI: 10.1107/S1600536809039269/tk2538Isup2.hkl

Additional supplementary materials:  crystallographic information; 3D view; checkCIF report

## Figures and Tables

**Fig. 1.**
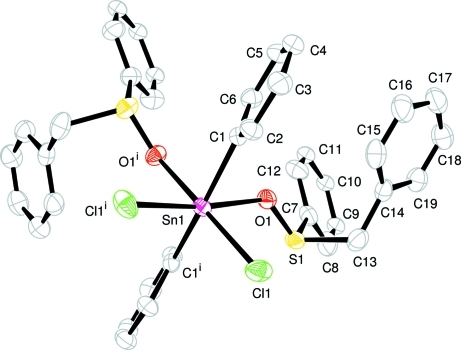
The structure of (I) with displacement ellipsoids are drawn at the 30% probability level. The H atoms have been omitted for clarity. The molecule has crystallographic twofold symmetry. Symmetry operation i: 2 - x, y, 1/2 - z.

## supplementary crystallographic information

### Comment

Organotin compounds have received considerable attention due to their structural diversity as well as due to their industrial, agricultural and biological applications (Davies, 2004; Hadjikakou & Hadjiliadis, 2009; Tian *et al.*, 2005). Several structures of organotin sulfoxide complexes, such as dichlorobis(dimethylsulfoxide-*O*)diphenyltin (Rehman *et al.*, 2007), dichlorodimethyl(dibenzylsulfoxide-O)tin (Ng & Rheingold, 1989), [bis(phenylsulfinyl)ethane-*O*,*O*]dichlorodiphenyltin (Boa *et al.*, 1995), and trichloro(dibutylsulfoxide)(ethoxycarbonylethyl)tin (Tian *et al.*, 1998) have been reported. As a continuation of these studies, the structure of the title compound, (I), is described herein.

The molecule of (I), Fig. 1, has crystallographic twofold symmetry. The Sn atom is six-coordinate within a distorted C_2_Cl_2_O_2_ octahedral geometry with *trans* phenyl groups, *cis* sulfoxides-O atoms, and *cis* chlorides. The Sn—C and Sn—Cl bond distances are similar to those found in dichlorobis(dimethylsulfoxide-*O*)diphenyltin (Rehman *et al.*, 2007), but the Sn—O length is longer. The C1—Sn1—C1^i^ and O1—Sn1—Cl^i^ angles are 162.98 (14) and 172.95 (5)°, respectively; i: 2 - *x*, *y*, 1/2 - *z*. The dihedral angle between the phenyl rings in the sulfoxide ligand is 46.8 (3)°.

### Experimental

Benzylphenylsulfoxide (0.865 g, 4 mmol) and diphenyltin dichloride (0.687 g, 2 mmol) in ethanol (30 ml) were refluxed for 1 h, and then the colourless solution was condensed and cooled. The solid product was filtered off and recrystallized from methanol. The colourless crystals suitable for X-ray analysis were obtained from the same solvent by slow evaporation (yield 72%; m.p. 383–384 K).

### Refinement

H atoms were placed at calculated positions with C—H = 0.93—0.93 Å, and refined in the riding model approximation with *U*_iso_(H) = 1.2*U*_eq_(C).

### Crystal data

**Table:**

[Sn(C_6_H_5_)_2_Cl_2_(C_13_H_12_OS)_2_]	*F*(000) = 1576
*M**_r_* = 776.40	*D*_x_ = 1.440 Mg m^−^^3^
Monoclinic, *C*2/*c*	Mo *K*α radiation, λ = 0.71073 Å
Hall symbol: -C 2yc	Cell parameters from 3086 reflections
*a* = 22.7485 (19) Å	θ = 2.7–24.9°
*b* = 11.5478 (14) Å	µ = 1.01 mm^−^^1^
*c* = 16.984 (2) Å	*T* = 295 K
β = 126.633 (6)°	Block, colourless
*V* = 3580.3 (7) Å^3^	0.25 × 0.22 × 0.11 mm
*Z* = 4	

### Data collection

**Table:**

Bruker SMART APEX area-detector diffractometer	3512 independent reflections
Radiation source: fine-focus sealed tube	3003 reflections with *I* > 2σ(*I*)
graphite	*R*_int_ = 0.031
φ and ω scans	θ_max_ = 26.0°, θ_min_ = 2.1°
Absorption correction: multi-scan (*SADABS*; Bruker, 2002)	*h* = −24→28
*T*_min_ = 0.786, *T*_max_ = 0.897	*k* = −14→12
9821 measured reflections	*l* = −20→20

### Refinement

**Table:**

Refinement on *F*^2^	Primary atom site location: structure-invariant direct methods
Least-squares matrix: full	Secondary atom site location: difference Fourier map
*R*[*F*^2^ > 2σ(*F*^2^)] = 0.032	Hydrogen site location: inferred from neighbouring sites
*wR*(*F*^2^) = 0.076	H-atom parameters constrained
*S* = 1.03	*w* = 1/[σ^2^(*F*_o_^2^) + (0.0324*P*)^2^ + 1.727*P*] where *P* = (*F*_o_^2^ + 2*F*_c_^2^)/3
3512 reflections	(Δ/σ)_max_ = 0.001
204 parameters	Δρ_max_ = 0.43 e Å^−^^3^
0 restraints	Δρ_min_ = −0.37 e Å^−^^3^

### Special details

**Table:**

Geometry. All e.s.d.'s (except the e.s.d. in the dihedral angle between two l.s. planes) are estimated using the full covariance matrix. The cell e.s.d.'s are taken into account individually in the estimation of e.s.d.'s in distances, angles and torsion angles; correlations between e.s.d.'s in cell parameters are only used when they are defined by crystal symmetry. An approximate (isotropic) treatment of cell e.s.d.'s is used for estimating e.s.d.'s involving l.s. planes.
Refinement. Refinement of *F*^2^ against ALL reflections. The weighted *R*-factor *wR* and goodness of fit *S* are based on *F*^2^, conventional *R*-factors *R* are based on *F*, with *F* set to zero for negative *F*^2^. The threshold expression of *F*^2^ > σ(*F*^2^) is used only for calculating *R*-factors(gt) *etc*. and is not relevant to the choice of reflections for refinement. *R*-factors based on *F*^2^ are statistically about twice as large as those based on *F*, and *R*- factors based on ALL data will be even larger.

### Fractional atomic coordinates and isotropic or equivalent isotropic displacement parameters (Å^2^)

**Table:**

	*x*	*y*	*z*	*U*_iso_*/*U*_eq_	
Sn1	1.0000	0.87434 (2)	0.2500	0.03980 (10)	
Cl1	0.90700 (5)	1.01568 (7)	0.21844 (6)	0.0683 (2)	
S1	0.84234 (4)	0.71126 (7)	0.15603 (5)	0.0544 (2)	
O1	0.92338 (10)	0.72512 (16)	0.23458 (13)	0.0499 (5)	
C1	1.04788 (14)	0.8471 (2)	0.40104 (18)	0.0396 (6)	
C2	1.06132 (16)	0.9415 (2)	0.45947 (19)	0.0489 (7)	
H2	1.0526	1.0158	0.4334	0.059*	
C3	1.08741 (18)	0.9272 (3)	0.5554 (2)	0.0600 (8)	
H3	1.0954	0.9916	0.5936	0.072*	
C4	1.10169 (17)	0.8184 (3)	0.5954 (2)	0.0589 (8)	
H4	1.1197	0.8089	0.6605	0.071*	
C5	1.08941 (18)	0.7241 (3)	0.5389 (2)	0.0585 (8)	
H5	1.0989	0.6502	0.5658	0.070*	
C6	1.06288 (16)	0.7378 (2)	0.4418 (2)	0.0493 (7)	
H6	1.0552	0.6732	0.4040	0.059*	
C7	0.82742 (17)	0.5595 (3)	0.1480 (2)	0.0558 (8)	
C8	0.7599 (2)	0.5161 (4)	0.0741 (3)	0.0748 (10)	
H8	0.7222	0.5655	0.0287	0.090*	
C9	0.7489 (3)	0.3981 (4)	0.0682 (4)	0.0967 (15)	
H9	0.7028	0.3680	0.0198	0.116*	
C10	0.8041 (3)	0.3252 (4)	0.1318 (4)	0.0978 (15)	
H10	0.7960	0.2457	0.1265	0.117*	
C11	0.8722 (3)	0.3686 (3)	0.2041 (3)	0.0867 (13)	
H11	0.9103	0.3184	0.2472	0.104*	
C12	0.8842 (2)	0.4863 (3)	0.2131 (2)	0.0645 (9)	
H12	0.9301	0.5161	0.2624	0.077*	
C13	0.79848 (19)	0.7551 (3)	0.2128 (3)	0.0685 (9)	
H13A	0.8046	0.8381	0.2236	0.082*	
H13B	0.7464	0.7400	0.1666	0.082*	
C14	0.82573 (17)	0.6978 (2)	0.3077 (2)	0.0537 (7)	
C15	0.8879 (2)	0.7370 (3)	0.3947 (3)	0.0699 (10)	
H15	0.9147	0.7972	0.3940	0.084*	
C16	0.9109 (2)	0.6882 (4)	0.4823 (3)	0.0849 (12)	
H16	0.9526	0.7160	0.5407	0.102*	
C17	0.8720 (2)	0.5978 (3)	0.4837 (3)	0.0789 (11)	
H17	0.8873	0.5646	0.5431	0.095*	
C18	0.8116 (2)	0.5575 (3)	0.3988 (3)	0.0715 (10)	
H18	0.7855	0.4963	0.4000	0.086*	
C19	0.7882 (2)	0.6056 (3)	0.3109 (3)	0.0637 (9)	
H19	0.7469	0.5762	0.2529	0.076*	

### Atomic displacement parameters (Å^2^)

**Table:**

	*U*^11^	*U*^22^	*U*^33^	*U*^12^	*U*^13^	*U*^23^
Sn1	0.04550 (17)	0.04045 (16)	0.03513 (15)	0.000	0.02496 (13)	0.000
Cl1	0.0833 (6)	0.0662 (5)	0.0587 (5)	0.0318 (4)	0.0441 (5)	0.0117 (4)
S1	0.0453 (4)	0.0608 (5)	0.0503 (4)	−0.0038 (3)	0.0249 (4)	0.0146 (3)
O1	0.0448 (11)	0.0542 (12)	0.0485 (11)	−0.0061 (9)	0.0266 (10)	0.0059 (9)
C1	0.0386 (15)	0.0442 (15)	0.0390 (14)	0.0034 (11)	0.0248 (13)	0.0039 (11)
C2	0.0582 (19)	0.0468 (16)	0.0419 (15)	0.0056 (14)	0.0301 (15)	0.0013 (12)
C3	0.062 (2)	0.070 (2)	0.0458 (17)	0.0032 (17)	0.0311 (16)	−0.0098 (16)
C4	0.0514 (19)	0.086 (2)	0.0390 (16)	0.0066 (17)	0.0267 (15)	0.0091 (16)
C5	0.061 (2)	0.0587 (19)	0.0521 (18)	0.0103 (15)	0.0313 (17)	0.0199 (15)
C6	0.0501 (17)	0.0461 (16)	0.0485 (16)	0.0052 (13)	0.0277 (15)	−0.0004 (13)
C7	0.059 (2)	0.065 (2)	0.0481 (17)	−0.0162 (16)	0.0346 (16)	−0.0010 (15)
C8	0.058 (2)	0.100 (3)	0.065 (2)	−0.026 (2)	0.0362 (19)	−0.015 (2)
C9	0.105 (4)	0.110 (4)	0.095 (3)	−0.063 (3)	0.070 (3)	−0.043 (3)
C10	0.156 (5)	0.071 (3)	0.108 (4)	−0.045 (3)	0.101 (4)	−0.023 (3)
C11	0.123 (4)	0.064 (2)	0.074 (3)	−0.012 (2)	0.060 (3)	0.0100 (19)
C12	0.074 (2)	0.058 (2)	0.0545 (19)	−0.0076 (17)	0.0350 (19)	0.0064 (16)
C13	0.058 (2)	0.059 (2)	0.095 (3)	0.0126 (16)	0.049 (2)	0.0185 (18)
C14	0.0533 (19)	0.0466 (17)	0.075 (2)	0.0061 (14)	0.0458 (18)	0.0034 (15)
C15	0.076 (2)	0.065 (2)	0.094 (3)	−0.0187 (18)	0.064 (2)	−0.019 (2)
C16	0.078 (3)	0.121 (3)	0.066 (2)	−0.013 (2)	0.049 (2)	−0.020 (2)
C17	0.097 (3)	0.086 (3)	0.080 (3)	0.005 (2)	0.067 (3)	0.005 (2)
C18	0.088 (3)	0.060 (2)	0.086 (3)	−0.0102 (19)	0.063 (2)	−0.0022 (19)
C19	0.063 (2)	0.058 (2)	0.077 (2)	−0.0051 (16)	0.046 (2)	−0.0049 (16)

### Geometric parameters (Å, °)

**Table:**

Sn1—C1	2.129 (2)	C8—H8	0.9300
Sn1—C1^i^	2.129 (2)	C9—C10	1.355 (7)
Sn1—O1	2.3519 (18)	C9—H9	0.9300
Sn1—O1^i^	2.3519 (18)	C10—C11	1.375 (6)
Sn1—Cl1^i^	2.4645 (8)	C10—H10	0.9300
Sn1—Cl1	2.4645 (8)	C11—C12	1.376 (4)
S1—O1	1.506 (2)	C11—H11	0.9300
S1—C7	1.775 (3)	C12—H12	0.9300
S1—C13	1.824 (3)	C13—C14	1.492 (4)
C1—C6	1.380 (4)	C13—H13A	0.9700
C1—C2	1.381 (4)	C13—H13B	0.9700
C2—C3	1.372 (4)	C14—C15	1.377 (5)
C2—H2	0.9300	C14—C19	1.386 (4)
C3—C4	1.372 (4)	C15—C16	1.370 (5)
C3—H3	0.9300	C15—H15	0.9300
C4—C5	1.364 (4)	C16—C17	1.379 (5)
C4—H4	0.9300	C16—H16	0.9300
C5—C6	1.387 (4)	C17—C18	1.349 (5)
C5—H5	0.9300	C17—H17	0.9300
C6—H6	0.9300	C18—C19	1.369 (5)
C7—C8	1.374 (4)	C18—H18	0.9300
C7—C12	1.379 (4)	C19—H19	0.9300
C8—C9	1.379 (5)		
			
C1—Sn1—C1^i^	162.98 (14)	C7—C8—C9	118.8 (4)
C1—Sn1—O1	80.68 (8)	C7—C8—H8	120.6
C1^i^—Sn1—O1	86.85 (8)	C9—C8—H8	120.6
C1—Sn1—O1^i^	86.85 (8)	C10—C9—C8	121.0 (4)
C1^i^—Sn1—O1^i^	80.68 (8)	C10—C9—H9	119.5
O1—Sn1—O1^i^	85.78 (10)	C8—C9—H9	119.5
C1—Sn1—Cl1^i^	94.61 (7)	C9—C10—C11	120.0 (4)
C1^i^—Sn1—Cl1^i^	96.64 (7)	C9—C10—H10	120.0
O1—Sn1—Cl1^i^	172.95 (5)	C11—C10—H10	120.0
O1^i^—Sn1—Cl1^i^	88.74 (6)	C10—C11—C12	120.2 (4)
C1—Sn1—Cl1	96.64 (7)	C10—C11—H11	119.9
C1^i^—Sn1—Cl1	94.61 (7)	C12—C11—H11	119.9
O1—Sn1—Cl1	88.74 (6)	C11—C12—C7	119.2 (4)
O1^i^—Sn1—Cl1	172.95 (5)	C11—C12—H12	120.4
Cl1^i^—Sn1—Cl1	97.05 (5)	C7—C12—H12	120.4
O1—S1—C7	104.46 (13)	C14—C13—S1	116.2 (2)
O1—S1—C13	105.59 (14)	C14—C13—H13A	108.2
C7—S1—C13	100.05 (14)	S1—C13—H13A	108.2
S1—O1—Sn1	127.62 (10)	C14—C13—H13B	108.2
C6—C1—C2	118.6 (2)	S1—C13—H13B	108.2
C6—C1—Sn1	122.35 (19)	H13A—C13—H13B	107.4
C2—C1—Sn1	118.93 (18)	C15—C14—C19	118.2 (3)
C3—C2—C1	120.8 (3)	C15—C14—C13	120.8 (3)
C3—C2—H2	119.6	C19—C14—C13	121.0 (3)
C1—C2—H2	119.6	C16—C15—C14	120.8 (3)
C4—C3—C2	120.3 (3)	C16—C15—H15	119.6
C4—C3—H3	119.9	C14—C15—H15	119.6
C2—C3—H3	119.9	C15—C16—C17	119.9 (4)
C5—C4—C3	119.7 (3)	C15—C16—H16	120.1
C5—C4—H4	120.2	C17—C16—H16	120.1
C3—C4—H4	120.2	C18—C17—C16	119.8 (3)
C4—C5—C6	120.4 (3)	C18—C17—H17	120.1
C4—C5—H5	119.8	C16—C17—H17	120.1
C6—C5—H5	119.8	C17—C18—C19	120.7 (3)
C1—C6—C5	120.2 (3)	C17—C18—H18	119.6
C1—C6—H6	119.9	C19—C18—H18	119.6
C5—C6—H6	119.9	C18—C19—C14	120.5 (3)
C8—C7—C12	120.8 (3)	C18—C19—H19	119.7
C8—C7—S1	119.2 (3)	C14—C19—H19	119.7
C12—C7—S1	119.9 (2)		
			
C7—S1—O1—Sn1	151.62 (14)	C13—S1—C7—C8	78.8 (3)
C13—S1—O1—Sn1	−103.37 (15)	O1—S1—C7—C12	5.1 (3)
C1—Sn1—O1—S1	143.25 (16)	C13—S1—C7—C12	−104.0 (3)
C1^i^—Sn1—O1—S1	−48.38 (15)	C12—C7—C8—C9	2.5 (5)
O1^i^—Sn1—O1—S1	−129.25 (17)	S1—C7—C8—C9	179.7 (3)
Cl1—Sn1—O1—S1	46.31 (14)	C7—C8—C9—C10	−2.3 (6)
O1—Sn1—C1—C6	45.7 (2)	C8—C9—C10—C11	0.6 (7)
O1^i^—Sn1—C1—C6	−40.5 (2)	C9—C10—C11—C12	0.9 (6)
Cl1^i^—Sn1—C1—C6	−129.0 (2)	C10—C11—C12—C7	−0.7 (6)
Cl1—Sn1—C1—C6	133.3 (2)	C8—C7—C12—C11	−1.0 (5)
C1^i^—Sn1—C1—C2	−174.6 (2)	S1—C7—C12—C11	−178.2 (3)
O1—Sn1—C1—C2	−131.2 (2)	O1—S1—C13—C14	−52.3 (3)
O1^i^—Sn1—C1—C2	142.6 (2)	C7—S1—C13—C14	55.9 (3)
Cl1^i^—Sn1—C1—C2	54.1 (2)	S1—C13—C14—C15	82.2 (4)
Cl1—Sn1—C1—C2	−43.6 (2)	S1—C13—C14—C19	−99.3 (3)
C6—C1—C2—C3	−1.5 (4)	C19—C14—C15—C16	−1.9 (5)
Sn1—C1—C2—C3	175.5 (2)	C13—C14—C15—C16	176.6 (3)
C1—C2—C3—C4	1.1 (5)	C14—C15—C16—C17	0.9 (6)
C2—C3—C4—C5	−0.4 (5)	C15—C16—C17—C18	0.2 (6)
C3—C4—C5—C6	0.2 (5)	C16—C17—C18—C19	−0.2 (6)
C2—C1—C6—C5	1.4 (4)	C17—C18—C19—C14	−0.9 (5)
Sn1—C1—C6—C5	−175.6 (2)	C15—C14—C19—C18	1.9 (5)
C4—C5—C6—C1	−0.7 (5)	C13—C14—C19—C18	−176.6 (3)
O1—S1—C7—C8	−172.1 (2)		

Symmetry codes: (i) −*x*+2, *y*, −*z*+1/2.
